# Motion-resistant three-wavelength spatial frequency domain imaging system with ambient light suppression using an 8-tap CMOS image sensor

**DOI:** 10.1117/1.JBO.29.1.016006

**Published:** 2024-01-18

**Authors:** Yu Feng, Chen Cao, Yuto Shimada, Keita Yasutomi, Shoji Kawahito, Gordon T. Kennedy, Anthony J. Durkin, Keiichiro Kagawa

**Affiliations:** a Shizuoka University, Graduate School of Integrated Science and Technology, Hamamatsu, Japan; b Shizuoka University, Research Institution of Electronics, Hamamatsu, Japan; c University of California, Irvine, Beckman Laser Institute, Irvine, California, United States; d University of California, Irvine, Biomedical Engineering Department, Irvine, California, United States

**Keywords:** spatial frequency domain imaging, diffuse optics, multispectral imaging, multi-tap complementary metal-oxide semiconductor image sensor

## Abstract

**Significance:**

We present a motion-resistant three-wavelength spatial frequency domain imaging (SFDI) system with ambient light suppression using an 8-tap complementary metal-oxide semiconductor (CMOS) image sensor (CIS) developed at Shizuoka University. The system addresses limitations in conventional SFDI systems, enabling reliable measurements in challenging imaging scenarios that are closer to real-world conditions.

**Aim:**

Our study demonstrates a three-wavelength SFDI system based on an 8-tap CIS. We demonstrate and evaluate the system’s capability of mitigating motion artifacts and ambient light bias through tissue phantom reflectance experiments and *in vivo* volar forearm experiments.

**Approach:**

We incorporated the Hilbert transform to reduce the required number of projected patterns per wavelength from three to two per spatial frequency. The 8-tap image sensor has eight charge storage diodes per pixel; therefore, simultaneous image acquisition of eight images based on multi-exposure is possible. Taking advantage of this feature, the sensor simultaneously acquires images for planar illumination, sinusoidal pattern projection at three wavelengths, and ambient light. The ambient light bias is eliminated by subtracting the ambient light image from the others. Motion artifacts are suppressed by reducing the exposure and projection time for each pattern while maintaining sufficient signal levels by repeating the exposure. The system is compared to a conventional SFDI system in tissue phantom experiments and then *in vivo* measurements of human volar forearms.

**Results:**

The 8-tap image sensor-based SFDI system achieved an acquisition rate of 9.4 frame sets per second, with three repeated exposures during each accumulation period. The diffuse reflectance maps of three different tissue phantoms using the conventional SFDI system and the 8-tap image sensor-based SFDI system showed good agreement except for high scattering phantoms. For the *in vivo* volar forearm measurements, our system successfully measured total hemoglobin concentration, tissue oxygen saturation, and reduced scattering coefficient maps of the subject during motion (16.5 cm/s) and under ambient light (28.9 lx), exhibiting fewer motion artifacts compared with the conventional SFDI.

**Conclusions:**

We demonstrated the potential for motion-resistant three-wavelength SFDI system with ambient light suppression using an 8-tap CIS.

## 1 Introduction

Monitoring tissue metabolic and perfusion information such as oxygenation and hemoglobin concentration plays a crucial role in healthcare and therapy. Traditional methods commonly used to obtain such information include using a pulse oximeter^1^ attached to the patient’s finger or employing near-infrared spectroscopy (NIRS)^2^ using probes. However, neither of them can provide noncontact measurements nor cover a wide field of view. Spatial frequency domain imaging (SFDI) is an imaging technique that projects structured light with different spatial frequencies and phases onto diffusely reflecting objects to quantify absorption and reduced scattering coefficients.^3^^,^^4^ SFDI is a noninvasive, noncontact, real-time, wide-field, quantitative imaging technique. Compact SFDI systems designed for bedside use have been employed in various experiments, including predicting diabetic foot ulcer,^5^^–^^7^ oxygenation measurement during reconstructive breast surgery,^8^ monitoring skin cancer,^9^ imaging and staging pressure ulcers,^10^ among others. SFDI is a particularly promising method for diagnosing burn severity since changes in reduced scattering measured using SFDI have been shown to correlate with histologically verified changes in collagen as a function of burn severity. These changes can be detected as early as 24 h after the burn.^11^^,^^12^ This change in scattering, along with tissue oxygen saturation (${\mathrm{StO}}_{2}$) and total hemoglobin concentration ($c_{\mathrm{tHB}}$) derived from absorption coefficients, enable clinicians to make improved treatment decisions and can sometimes lead to early recovery for the patient.^13^ To accurately quantify tissue metabolic information, particularly the concentrations of oxy/deoxy hemoglobin, we require SFDI measurements at two or more wavelengths. However, since the presence of melanin in the epidermis can complicate the measurement results of other tissue chromophores, at least three wavelengths (as many as the number of chromophores) are generally required to achieve accurate determination of oxy and deoxyhemoglobin concentrations.

One of the limitations of SFDI is its susceptibility to ambient light.^14^ Most state-of-the-art SFDI systems require operating in low-light or dark conditions, as ambient light becomes a bias for the pattern projection and consumes detector dynamic range. However, there is an increasing demand for SFDI systems to be used in a variety of environments, including bright environments such as patient rooms, operating rooms, and outdoors. In these scenarios, the ambient light bias poses a challenge for accurate SFDI measurements.

Another challenge in SFDI is motion artifacts, which can be caused by the movement of the sample region of interest (ROI) during sequential pattern projection (i.e., motion due to respiratory cycles and shaking due to pain). As we assume that the captured raw images are stationary in the subsequent image processing, motion artifacts appear in the measurement results.^14^ They tend to be most pronounced at the subject’s edges and in regions with substantial variations in absorption and reduced scattering coefficients, such as in proximity to blood vessels. These artifacts, in turn, introduce errors into the measured skin optical properties.

In this study, to address these limitations, we present the development of a three-wavelength SFDI system based on an 8-tap complementary metal-oxide semiconductor (CMOS) image sensor (CIS) prototype. Our system offers a unique ability to simultaneously measure three wavelength bands, effectively addressing two prominent challenges faced by conventional SFDI systems: ambient light bias and motion artifacts. Specifically, we demonstrate the system’s efficacy in measuring the volar forearm of two human subjects under ambient light conditions and during controlled motion of the subjects’ arm. These two subjects were selected because their blood vessels are more visible, enabling easier assessment of any motion artifacts that may occur.

## 2 Materials and Methods

### 2.1 Multitap CIS

The multitap CIS pixel is composed of a single photodiode (PD), and multiple pairs of charge transfer gates (TGs) and storage diodes (SDs), referred to as “taps.” This unique design enables the multitap image sensor pixel to output multiple pixel values upon readout, as opposed to the single pixel value output from a typical image sensor pixel. Schwarte et al.^15^ first introduced the original 2-tap pixel based on photogates. 4-tap pixels have been developed by Seo et al.^16^ from our research group utilizing the lateral electric field charge modulator (LEFM)^17^ and by Keel et al.,^18^ who employed the photogate. Hatakeyama et al.^19^ and Kuo and Kuroda^20^ developed 4-tap pixels using TGs. Our research group successfully developed an 8-tap pixel utilizing the LEFM technology, specifically for the purpose of short-pulse-based time-of-flight range measurements.^21^

**Fig. 1:**
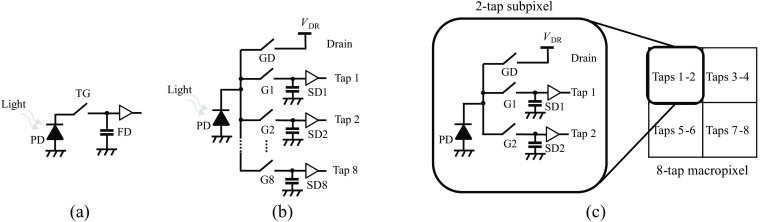
(a) Simplified schematic diagram of a typical image sensor pixel (reset switch is omitted), (b) an 8-tap image sensor pixel, (c) and an 8-tap macropixel with a 2×2-subpixel configuration.

Figures 1(a) and 1(b) depict the structures of a typical image sensor pixel and an 8-tap sensor pixel, respectively. The charge TGs (G1 to G8) modulate the flow of the photogenerated charges, whereas the SD temporarily stores the transferred charges. In typical image sensor pixels, the TG turns on and transfers the photogenerated charges into the floating diffusion (FD) after the accumulation time. In contrast, in an 8-tap sensor, a designated TG among G1 to G8 turns on, and transfers the photogenerated charges to the corresponding SD. The gate for draining charges (GD) is used to make an insensitive period to the incident light, e.g., during the image readout. Subsequently, during the readout time, a typical image sensor pixel outputs a single pixel value, whereas an 8-tap image sensor pixel simultaneously outputs eight pixel values. Moreover, the 8-tap image sensor pixel allows multiple exposures, as the TG can be turned on and off multiple times during the accumulation time.

In this study, we utilize a laboratory-designed 8-tap CIS pixel based on LEFM technology. Compared with the original 8-tap image sensor,^21^ the sensor features a smaller pixel size and a higher fill factor. Note that the pixel array of this image sensor is composed of macropixels with a 2×2-subpixel configuration, where each subpixel consists of two taps and a drain, as illustrated in Fig. 1(c). The layout of the subpixel cell is similar to the reported 2-tap pixel.^22^ Thus, it can be defined as a “virtual” 8-tap pixel. The pixel design complexity is significantly reduced compared with the original 8-tap sensor. The specifications of this virtual 8-tap CIS are briefly shown in Table 1.

**Table 1:** Sensor specifications.

Parameters	Value
Macropixel size (${\mu m}^{2}$)	12.6 × 12.6
Subpixel number/macropixel	2 × 2
Macropixel number	700 (H) × 540 (V)
Tap number/subpixel	2 taps + drain
Max. frame rate (fps)	33

Despite the native resolution of 700×540, the 8-tap sensor’s macropixel configuration, followed by interpolation similar to that used in RGB cameras, results in an effective resolution of 1400 × 1080 pixels. This effective resolution is comparable with typical cameras used in SFDI. The prototyped image sensor is based on 0.11  μm CIS process, resulting in relatively large pixel size, about $12.6\times 12.6\;{\mu m}^{2}$. However, when a more advanced CIS process designed for mass production is available, the pixel size can be reduced, allowing more pixels within the same pixel area. While the taps and drains of the multitap sensor will still require some overhead for pixel shrinkage compared to a normal sensor, the overall functionality it provides remains beneficial.

### 2.2 System Operation

**Fig. 2:**
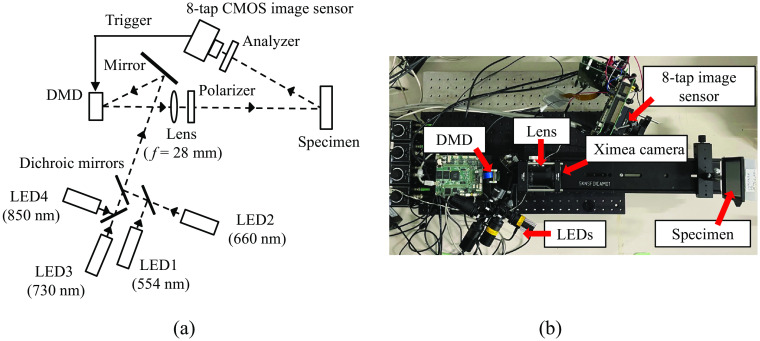
(a) Schematic diagram of the SFDI system. (b) A photograph of the system (top view).

Figure 2 shows the schematic diagram and a photograph of the SFDI system utilized in this study. The working distance of the 8-tap system is 24 cm. The system employs light-emitting diodes (LEDs) with wavelengths of 660, 730, and 850 nm to quantify oxyhemoglobin and deoxyhemoglobin, and the reduced scattering coefficients on a pixel-by-pixel basis. A two-dimensional sinusoidal patterned light is generated by a digital micromirror device (DMD; Keynote Photonics, LC4500NIR) and projected onto the sample. The reflected light is then captured by the 8-tap image sensor. During the imaging process, after the exposure for one pattern is finished, the field-programmable gate array (FPGA) board that controls the sensor sends a trigger signal to the DMD, initiating the projection of the next pattern. Most conventional SFDI systems utilize a sinusoidal pattern at a particular spatial frequency, projected three times, each time shifted by 2π/3 radians for each wavelength. However, in this study, we employ an improved acquisition method based on the Hilbert transform,^23^ which reduces the number of required images per wavelength (for a particular spatial frequency) from three to two, i.e., an image for planar illumination (direct current (DC) or sine wave at $0\;{\mathrm{mm}}^{-1}$) and that for a single sinusoidal pattern (alternate current (AC) or sine wave at $0.1\;{\mathrm{mm}}^{-1}$). In this study, we capture DC and AC images at the three wavelengths simultaneously using the 8-tap image sensor. Through the application of the Hilbert transform, we calculated the corresponding cosine image from the sine image. Additionally, we capture an image only for the ambient light. Since there is one spare tap available, a DC image for a wavelength (554 nm, LED) is captured. This wavelength is suitable for separating the melanin present in the epidermis. However, at the present stage, this additional DC image is not utilized in the subsequent processes but will be used in future work.

**Fig. 3:**
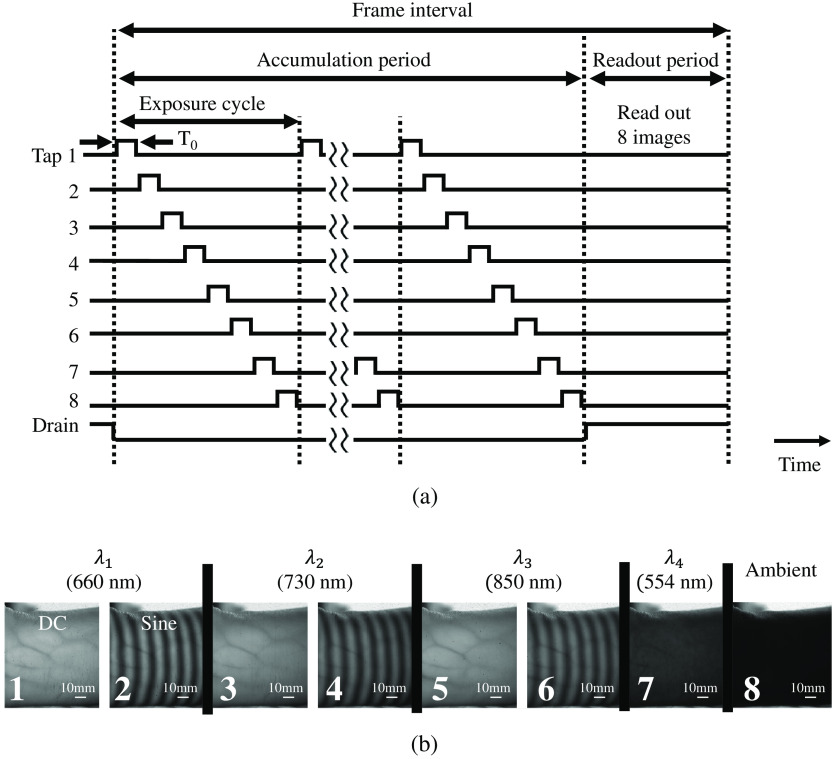
(a) Timing chart of the 8-tap image sensor operation. (b) An example of captured raw images of the 8-tap image sensor-based SFDI system under ambient light.

Figure 3(a) illustrates the timing chart of the 8-tap sensor operation. In this chart, $T_{0}$ represents the exposure time for each tap within an exposure cycle. In this work, to mitigate motion artifacts, the time for each pattern projection and sensor exposure is shortened to reduce time lag and make any motion among the frames imperceptible. During the accumulation period, the exposure cycle repeats *N* times to ensure that the brightness of the captured images from each tap is maintained. The total exposure time for each tap is denoted as N $T_{0}$. Unlike conventional high-speed image sensors that read out each image after each exposure, the 8-tap sensor reads out only after the accumulation period finishes, outputting all eight images simultaneously in the readout period.

This unique multi-exposure capability results in fewer readouts. Although high-frame-rate image sensors can emulate the operation of multitap image sensors, they have drawbacks such as higher total read noise, heat dissipation, and processing power. When the number of exposure cycles is three and the image readout frame rate is 9.4 fps, which is used in the experiments below, the frame rate for equivalent high-frame-rate image sensors becomes 225.6 fps. Higher frame rates lead to higher heat dissipation leading to shorter battery-operating time and difficulty in miniaturization of the camera. Furthermore, higher computation power is required to process more images. Noise also is of significant concern. Because the readout noise is added to every frame, the total read noise becomes roughly three times higher than that of the multitap image sensor. The 8-tap sensor’s capability is particularly valuable in scenarios that do not require high frame rates, such as measuring the optical properties of tissue, where they are affected by respiratory cycles or shaking due to pain. In such scenarios, the 8-tap sensor can provide sufficient frame rates while enabling motion artifacts mitigation.

Figure 3(b) shows an example of the captured raw images. The numbers displayed on each image indicate the corresponding tap index. By subtracting the ambient light image from the other images, the ambient light bias is eliminated. The exposure time $T_{0}$ is set to 3 ms for each pattern due to the minimum projection time limitation of the DMD for 6-bit grayscale patterns. The exposure cycle was repeated three times to maintain the brightness of the captured images. The total exposure time for each tap is 9 ms. The 8-tap SFDI system operates at a frame rate of 9.4 fps, with each frame set comprising eight images. The frame interval, as shown in Fig. 3(a), includes both the accumulation period and the subsequent readout period.

### 2.3 SFDI

As described in Ref. 3, the projected AC pattern, denoted as S, is represented as

$$S=\frac{S_{0}}{2}[1+M_{0}\cos(2\pi f_{x}x+\phi )],$$ (1)

where $S_{0}$, $M_{0}$, $f_{x}$, and ϕ represent the intensity of the illumination source, modulation depth, spatial frequency, and spatial phase, respectively.

The reflected intensity, denoted as I, consists of a DC component $I_{\mathrm{DC}}$ and an AC component $I_{\mathrm{AC}}$, which is expressed as

$$I(x,f_{x})=I_{\mathrm{DC}}(x)+I_{\mathrm{AC}}(x,f_{x})=\alpha_{\mathrm{DC}}M_{\mathrm{DC}}(x)+\alpha_{\mathrm{AC}}M_{\mathrm{AC}}(x,f_{x})\cdot \cos(2\pi f_{x}x+\phi ),$$ (2)

where, $M_{\mathrm{DC}}(x)$ represents the DC reflectance at a position x, and $M_{\mathrm{AC}}(x,f_{x})$ represents the AC reflectance of spatial frequency $f_{x}$ at the position x. $\alpha_{\mathrm{DC}}$ and $\alpha_{\mathrm{AC}}$ are coefficients to convert the reflectance to the pixel value, which are removed in the calibration stage. For the acquisition of reflectance, we utilize the advanced SFDI acquisition method described in Ref. 23. This method sequentially projects one DC pattern and one AC pattern with a phase offset ϕ of 0 onto the sample. The AC pattern with the phase offset ϕ of *π*/2 radians is calculated by using the Hilbert transform. The DC and AC reflectance, $M_{\mathrm{DC}}(x)$ and $M_{\mathrm{AC}}(x,f_{x})$, are then calculated at the position x as follows:

$$M_{\mathrm{DC}}(x)=(1/\alpha_{\mathrm{DC}})I_{\mathrm{DC}}(x),$$ (3)

$$M_{\mathrm{AC}}(x,f_{x})=\;(1/\alpha_{\mathrm{AC}})\sqrt{(I_{0}(x)-I_{\mathrm{DC}}(x){)}^{2}+(I_{\frac{\pi }{2}}(x)-I_{\mathrm{DC}}(x){)}^{2}},$$ (4)

where $I_{\mathrm{DC}}(x)$, $I_{0}(x)$, and $I_{\frac{\pi }{2}}(x)$ are the pixel values of the DC, the AC for the phase of 0, and the AC for the phase of π/2 radians, respectively. Due to the 2×2-subpixel configuration of the pixel, raw images are interpolated for each tap using the MATLAB function “interp2.”

Since the measured result considers factors such as the intensity of the light source and the *f*-number of the lens, among others, to obtain the sample’s reflectance, the measured results are calibrated using a tissue-mimicking phantom with known optical properties as a reference measurement.

Theoretically, the DC components of the AC image should be half of that of the corresponding DC image. However, it should be noted that the measured AC image’s DC component may not necessarily equal half of the DC component of the DC image, due to the differences in sensitivity among different taps of the multitap sensor pixel. The variation in the DC components introduces errors during the process of subtracting the DC images from the AC images, as Eq. (4) suggests. These errors can lead to residual sinusoidal patterns in the resulting $M_{\mathrm{AC}}$ image.

**Fig. 4:**
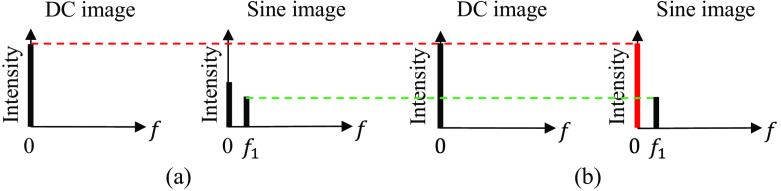
The intensity of frequency components in the DC and sine images: (a) before and (b) after DC compensation.

To address this issue, a Fourier transform is performed on each set of input images for the reference measurement. Figure 4(a) shows the intensity of frequency components in the DC and sine images. Within each set of the DC image and sine image, the intensity of the DC components (i.e., the intensity at $f=0\;{\mathrm{mm}}^{-1}$) are different. The coefficient defined by the DC component in the DC image divided by the DC component in the sine image is used to equalize the DC components. The resulting intensity of frequency components after DC compensation is shown in Fig. 4(b). Note that the AC component in the sine image remains unchanged during this processing. The calibration to convert the pixel value to the reflectance $R_{\mathrm{DC}}$ and $R_{\mathrm{AC}}$ is applied after this processing.

Using the true (calibrated) reflectance $R_{\mathrm{DC}}$ and $R_{\mathrm{AC}}$ at each wavelength, oxy/deoxyhemoglobin concentrations, and scattering parameters a and b are determined by referring to a precalculated look-up table (LUT). The scattering parameters *a* and *b* are denoted as

$$\mu_{s}^{\prime }(\lambda )=a(\frac{\lambda }{500\;\mathrm{nm}}{)}^{-b},$$ (5)

where $\mu_{s}^{\prime }$ is the wavelength dependent reduced scattering coefficient, a is the scattering amplitude normalized to 500 nm, and *b* is the scattering power.

In this work, we assume a skin model with a semi-infinite slab geometry with homogeneously distributed optical properties and assumes the absence of melanin for simplicity. The LUT is generated using the Monte Carlo simulation based on the model. Both the concentrations of chromophores and scattering parameters are considered in the simulation model and the Monte Carlo simulation then calculates the reflectance values, $R_{\mathrm{DC}}$ and $R_{AC}$, at each wavelength for the combinations of the input parameters for given spatial frequencies. Table 2 shows the upper and lower bounds for the input parameters. The lower and upper bounds for the parameters are determined based on previous work^24^^–^^26^ while ensuring that they cover the range relevant to the subjects participating in the experiments.

**Table 2:** Parameters for LUT generation.

Parameter	Lower bound	Upper bound
a (${\mathrm{mm}}^{-1}$)	2.1	8.4
b	1	3
$c_{\mathrm{tHb}}\;(\mu M)$	4.6	300
${\mathrm{StO}}_{2}\;(\%)$	20	80

## 3 Results

To evaluate the performance of our 8-tap image-sensor-based system, we initially performed a side-by-side comparison with a conventional SFDI system based on three-phase demodulation and a commercial image sensor (Ximea, MQ013RG-E2, 1280 × 1024 pixels, 10 bit). The Ximea camera is placed under the lens, as shown in Fig. 2(b). The working distance of the Ximea system is 26 cm. The spatial frequency of the projected AC patterns was 0.10 mm^−1^. We compared the diffuse reflectance maps obtained using both systems for three tissue-mimicking phantoms with known optical properties ($\mu_{a}$ and $\mu_{s}^{\prime }$). We then performed *in vivo* volar forearm experiments under both the bright (ambient light intensity of 28.9 lx) and the dark (no ambient light) conditions. The ambient light is introduced by a fluorescent lamp, which contains little near-infrared light. In the *in vivo* volar forearm experiments where the subject’s arm remained stationary, we compared the chromophore concentrations and scattering parameter maps obtained by both systems. Finally, under ambient light while the subject’s arm was in motion, we compared videos of the chromophore concentrations and scattering parameter maps acquired by the 8-tap image sensor-based system. Specifically, we examined two configurations: one with a single exposure and another with shorter exposures repeated three times.

### 3.1 Tissue Phantom Reflectance Experiment

First, the diffuse reflectance maps of three tissue-mimicking phantoms were measured using both the 8-tap SFDI system and the Ximea SFDI system. The three phantoms used in this study are referred to as the 2-tone, gum, and skin phantoms. Only for the reference phantom, a complete table of the absorption and reduced scattering coefficients for a wavelength range of 450 to 1000 nm was available, which was measured by an integrating sphere system.^27^ The tissue-mimicking phantoms are made from silicone polymer, Black India Ink, and titanium-dioxide powder. The gum and skin phantoms have uniform optical properties. The 2-tone phantom has two distinct halves, each having different optical properties.

The measured optical properties of these phantoms, except for the reference phantom, were obtained using time-resolved NIRS equipment (TRS-20 or 80, Hamamatsu Photonics K.K., Japan) at either 797 or 801 nm. The specific values for these optical properties are shown in Table 3.

**Table 3:** Measured optical properties of phantoms using TRS (except “Reference”).

Phantom (wavelength)	$\mu_{a}$ (${\mathrm{mm}}^{-1}$)	$\mu_{s}^{\prime }$ (${\mathrm{mm}}^{-1}$)
Reference (800 nm)	0.0215	0.5576
2-Tone-dark (797 nm)	0.0487	0.8040
2-Tone-bright (797 nm)	0.0195	0.6038
Gum (801 nm)	0.0292	1.0875
Skin (801 nm)	0.0074	1.168

**Fig. 5:**
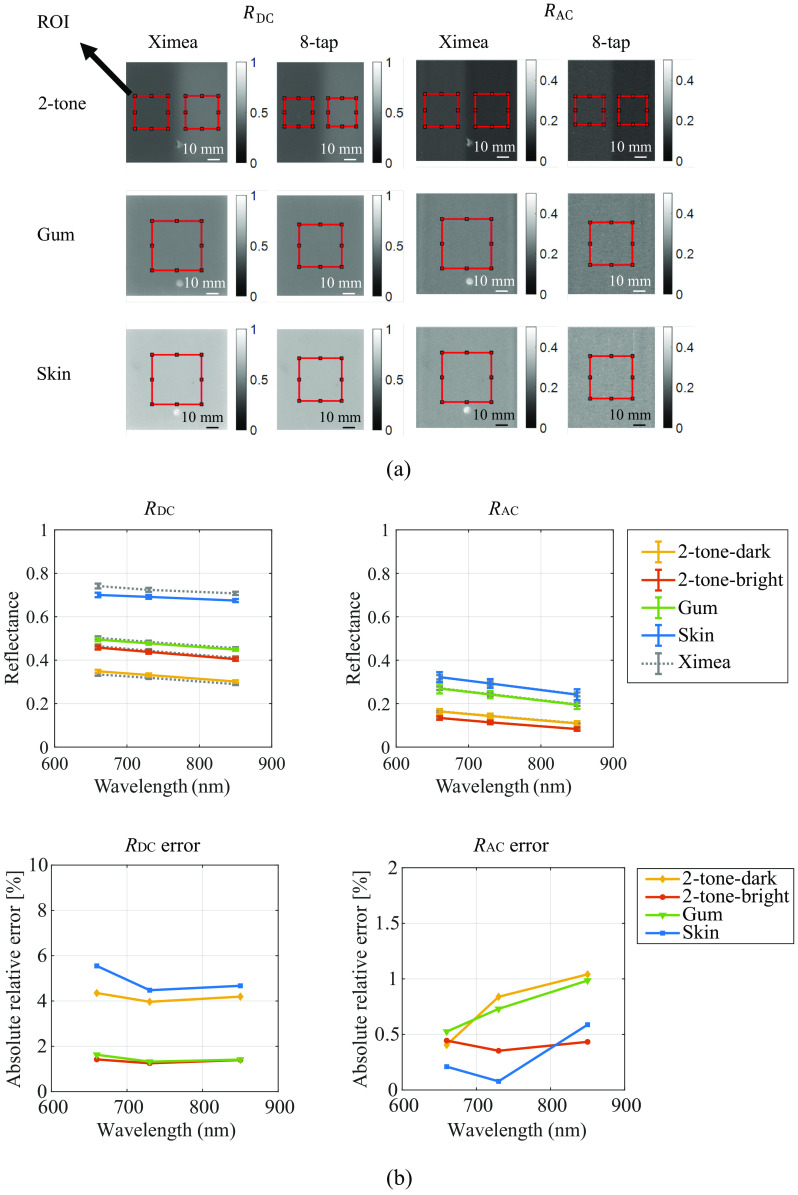
(a) Measured reflectance images of the phantoms, (b) the average reflectance’s DC and AC components within the ROI, and (c) the absolute relative error between the results of the two systems.

The exposure time for the Ximea system was 20 ms, whereas that for the 8-tap system was 9 ms (3 ms exposure repeated three times or 9 ms single exposure). To suppress the random readout noise and dark current shot noise, both systems averaged 10 sets of raw images during acquisition before performing image processing. The measured reflectance maps of the phantoms are compared in Figs. 5(a) and 5(b). The top two graphs in Fig. 5(b) show the mean reflectance for the DC component $R_{\mathrm{DC}}$, and the AC component $R_{\mathrm{AC}}$, within the ROI in Fig. 5(a). The size of the ROI is 200 × 200 pixels ($∼23\times 23\;{\mathrm{mm}}^{2}$) for the 2-tone phantoms and 300 × 300 pixels ($∼34\times 34\;{\mathrm{mm}}^{2}$) for other phantoms. Error bars represent the standard deviation. The bottom two graphs in Fig. 5(b) show the absolute relative error of the measurement results by the 8-tap system compared with those by the Ximea SFDI system. The measurements of the 8-tap system agreed with the Ximea system within ±1% for $R_{\mathrm{AC}}$ and ±6% for $R_{\mathrm{DC}}$. The measurements for the 8-tap system showed a slightly larger standard deviation, which can be caused by the higher noise of the 8-tap image sensor compared with the Ximea system. This is a limitation of our current prototype image sensor.

**Fig. 6:**
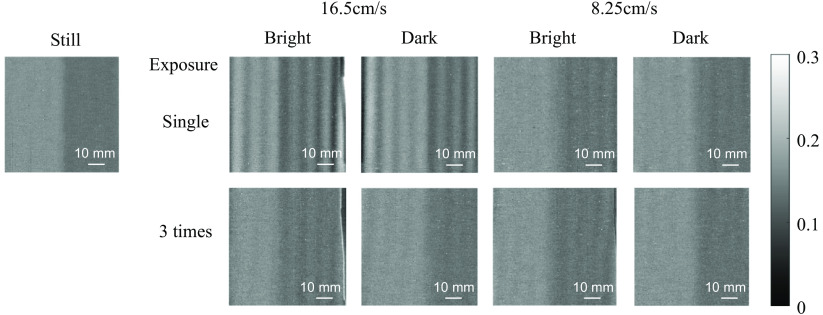
Measured AC components (660 nm) of the reflectance of the 2-tone phantom (bright: with ambient light of 28.9 lx, dark: no ambient light).

To validate the motion artifact suppression capability of our 8-tap system, we conducted experiments using a 2-tone phantom while it was under the influence of motion via a mechanical slider to characterize sensitivity to motion artifacts. The slider was set to move at speeds of ∼16.5 and 8.25 cm/s. We are trying to mitigate motion associated with pain and tremor, which can be part of what the burn patient experiences, particularly while trying to remain motionless during imaging. While we have not been able to identify literature that quantifies that motion, we have been able to identify literature related to Parkinson’s disease tremor and have used this as a surrogate target. We surveyed articles on Parkinson’s disease tremor and found that most Parkinson’s disease tremors have a typical frequency of 4 to 6 Hz.^28^^,^^29^ Assuming the tremor motion is sinusoidally oscillating with a peak-to-peak swing of 1 cm based on the supplementary online video in Ref. 29, the maximum speed of Parkinson’s disease tremor is calculated to be 12.6 to 18.8 cm/s. In addition, we require the technology to be useful across species, including preclinical experiments. Typically, these investigations involve animal subjects under anesthesia. However, motion related to respiration can confound data analysis. For rodents, respiration rates may be as high as 240 breaths/min.^30^ Thus, the results that we have demonstrated here are relevant to investigations using preclinical animal models. Additional application areas may include studies of cerebral hemodynamics in preclinical models.^31^ The slider moved in parallel to the direction of the wave vector of the projected AC patterns, moving from right to left on the image. We aimed to evaluate the system’s ability to suppress the effects of pain-induced movement without physically restraining the subjects. The experiments aimed to compare the effects of long single exposure (9 ms) performed in the conventional SFDI with three times repeated short exposure (3 ms × 3) under two different lighting conditions: bright (ambient light intensity of 28.9 lx) and dark (no ambient light) conditions. As an example, Fig. 6 displays the measured $R_{\mathrm{AC}}$ (660 nm) images. The results obtained using the 8-tap system, with the exposure repeated three times, demonstrated a noticeable reduction or absence of motion artifacts. In contrast, the single exposure results exhibited more pronounced motion artifacts, especially for the faster motion, resulting in residual sinusoidal patterns in the images. The $R_{\mathrm{AC}}$ images at the other two wavelengths also showed similar behaviors. This experiment confirmed the superior performance of our 8-tap system in terms of its resistance to motion artifacts, thereby providing more reliable and accurate measurements in dynamic situations.

### 3.2 *In Vivo* Volar Forearm Experiment

**Fig. 7:**
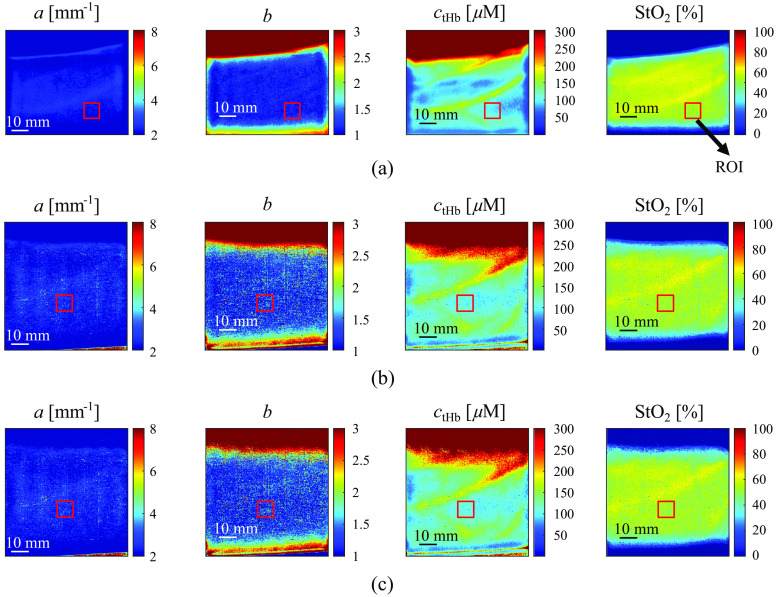
Obtained $a,b,c_{\mathrm{tHb}}$, and ${\mathrm{StO}}_{2}$ maps of subject #1’s volar forearm: (a) Ximea dark, (b) 8-tap dark, and (c) 8-tap bright.

**Fig. 8:**
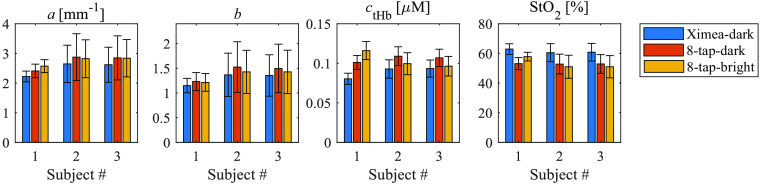
The average $a,b,c_{\mathrm{tHb}}$, and ${\mathrm{StO}}_{2}$ values in the ROI of the three subjects.

In*in vivo* volar forearm experiments, three healthy individuals (Asian males in their 20s) were recruited to participate. Figure 7 shows the $a,b,c_{\mathrm{tHb}}$, and ${\mathrm{StO}}_{2}$ maps obtained from the volar forearm of the subjects in dark condition (no ambient light) using both the Ximea SFDI system and our 8-tap SFDI system. The lighting, image acquisition, and image averaging conditions were the same as those in Sec. 3.1. The $a,b,c_{\mathrm{tHb}}$, and ${\mathrm{StO}}_{2}$ maps were generated using the LUT method described in Sec. 2.3. Figure 8 shows the average values of $a,b,c_{\mathrm{tHb}}$, and ${\mathrm{StO}}_{2}$ within the ROI of the two systems in the dark condition as well as the 8-tap system in the bright conditions. The error bars represent the standard deviation. The size of the ROI is 80 × 80 pixels ($∼9\;\times 9\;{\mathrm{mm}}^{2}$). Table 4 displays the relative errors of the 8-tap system in the dark and bright conditions, compared with the Ximea system in dark conditions. In the dark conditions, it shows that the a and b values in the ROI were relatively close for the two systems. However, the $c_{\mathrm{tHb}}$ and ${\mathrm{StO}}_{2}$ values exhibited a larger difference between the two systems. When comparing the 8-tap system in both the dark and bright conditions, the results indicate that the values of a,b, $c_{\mathrm{tHb}}$, and ${\mathrm{StO}}_{2}$ values in the ROI showed good agreement. These results confirmed that the 8-tap SFDI system successfully eliminated the bias caused by the ambient light. The standard deviation of the parameter *a* and *b* are relatively large. This might be because *a* and *b* related to the exponential function are susceptive to noise.

**Table 4:** Relative errors (%) of the 8-tap system in two conditions based on Ximea-dark.

System	Subject #	a	b	$c_{\mathrm{tHb}}$	${\mathrm{StO}}_{2}$
8-Tap-dark	1	8.41	7.06	25.78	−15.57
	2	8.61	11.51	17.38	−12.69
	3	8.85	10.45	14.45	−13.02
8-Tap-bright	1	15.65	5.56	44.31	−8.08
	2	6.62	4.44	7.22	−15.68
	3	8.33	5.34	3.14	−16.11

**Fig. 9:**
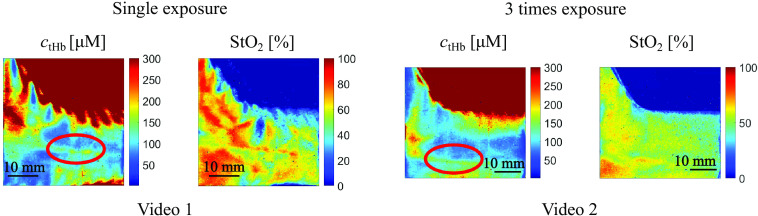
Extracted frames from Videos 1 and 2. Obtained $c_{\mathrm{tHb}}$ and ${\mathrm{StO}}_{2}$ maps of subject #1’s volar forearm in bright condition: without repeated exposure (Video 1) and with exposures repeated three times (Video 2).

Subsequently, *in vivo* volar forearm experiments during motion were performed with subject #1 and subject #2. In this work, only results of subject #1 are shown because the results of subject #2 exhibited the same trend as those of subject #1. The subject’s volar forearm was measured while the arm was placed on a mechanical slider, which was manually translated by the subject. The subjects moved the slider, and the total time was measured with a stopwatch. The measured speed of the slider represents the average speed. The subjects underwent several training sessions to ensure that the slider moved at a consistent speed during the measurements. The slider moved at a speed of ∼16.5  cm/s. The moving direction of the slider is the same as that in Sec. 3.1. The experiments were performed under two conditions: one with a single exposure and the other with exposures repeated three times, both in the bright condition (ambient light intensity of 28.9 lx). Videos 1 and 2 compare the estimated $c_{\mathrm{tHb}}$ and ${\mathrm{StO}}_{2}$ maps of the subject’s volar forearm during motion for the long single exposure and repeated exposure, respectively. The results shown in Fig. 9 are single frames extracted from the corresponding videos. The results indicate that the proposed method exhibits a reduction in motion artifacts compared with one with a single long exposure (conventional SFDI), especially in the region highlighted by the red circle. This demonstrates that our prototype 8-tap image sensor-based SFDI system is suitable for measuring subjects during motion under ambient light.

## 4 Discussion

In the tissue phantom reflectance experiment results shown in Fig. 5(b), it can be observed that the error of $R_{\mathrm{DC}}$ between the two systems is relatively high for the skin phantom. Similarly, in the *in vivo* volar forearm experiment results shown in Fig. 8, the average $a,b,c_{\mathrm{tHb}}$, and ${\mathrm{StO}}_{2}$ values for the three subjects also exhibit substantial discrepancies between the two systems. These disparities are potentially due to the skin and the skin phantom’s high scattering characteristics when compared with the reference phantom, as indicated in Table 3. Additionally, it should be noted that the capture angle for the 8-tap system is measured at ∼22.68  deg due to the bulky size of the FPGA board in the 8-tap system, whereas the capture angle for the Ximea system is ∼0  deg. The difference in capture angle may also contribute to the observed differences between the two systems.

It should be noted that the proposed method has not been verified with layered heterogeneous phantoms with known optical parameters. The experimental results in Fig. 6 only show that motion artifacts are suppressed for a single-layered heterogeneous (side-by-side) phantom. Although Fig. 9 implies that the proposed method could be also effective for layered heterogeneous tissues, it is important to verify the efficacy of the proposed method with layered heterogeneous phantoms mimicking skin with underlying blood vessels in future work.

Despite its advantages in motion resistance and ambient light bias suppression (ambient light intensity of 28.9 lx), our prototype 8-tap image sensor-based SFDI system exhibits a degradation in signal-to-noise ratio (SNR) compared with the conventional Ximea system. This degradation can be attributed to the design and operation of the sensor, as described in Ref. 22. The sensor in our system is specifically designed to detect short pulse light within consecutive time windows of 500 ns. This allows charges generated by ambient light and dark current to be effectively drained between time windows, leading to improved shot noise performance, and hence, the SNR in the system. However, in the case of our current 8-tap SFDI system, we used a 9 ms continuous-wave LED light source and noticeably short drain time, which does not adequately drain the charges generated by ambient light and dark current, resulting in a decrease in SNR. Acknowledging this problem, we limited our SFDI experiments to low ambient light conditions due to long LED emission time in this study. However, we suggest that our 8-tap SFDI system can overcome this limitation by employing a low duty ratio short pulse light source, which emits high-power light in a narrow pulse width (e.g., pulse with: a few μs) periodically, whereas it turns off after each illumination and remains off for most of the time, as demonstrated in Ref. 22. In future work, we will focus on investigating the implementation of low duty ratio short pulse light sources and extending the drain time to meet with the sensor’s specification. By incorporating these improvements, we expect to increase the SNR of the system and achieve higher quality measurements under more realistic ambient conditions. By addressing the SNR limitation, our prototype 8-tap image sensor-based SFDI system will become even more robust and reliable, further expanding its potential applications in various practical and versatile scenarios.

## 5 Conclusion

In this work, we have demonstrated a prototype three-wavelength SFDI system that is capable of accurately measuring optical properties in a ROI that is subject to motion under ambient light. This is done using a unique 8-tap CIS developed in our laboratory. The Hilbert transform enabled faster and more efficient data acquisition in the SFDI system by reducing the required number of projected patterns per wavelength from three to two per spatial frequency. The system was validated by the reflectance results in phantom experiments and the tissue optical properties results in the *in vivo* volar forearm experiments. The phantom experiment during motion also confirms the system’s ability to suppress motion artifacts. The 8-tap system’s results in bright and dark conditions were compared to a reference system based on a commercial sensor and three-phase SFDI in the dark condition. The results showed good agreement in most phantom experiments, but differences were higher for skin phantoms and volar forearm measurements with high scattering coefficients. Scattering parameters *a* and *b* showed larger standard variation than other tissue parameters probably because they are susceptive to the image sensor noise. *In vivo* volar forearm experiments during motion were performed on subject #1 and #2. Our system effectively measured moving subjects (16.5 cm/s) under ambient light (28.9 lx) without significant motion artifacts. Overall, this work demonstrates the feasibility and potential of our motion-resistant and ambient light-resistant SFDI system, which can provide enhanced imaging capabilities for accurate measurements in real-world clinical settings.

## 6 Appendix: Supplementary Information

**Video 1.** Obtained $c_{\mathrm{tHb}}$ and ${\mathrm{StO}}_{2}$ maps video of subject #1’s volar forearm in bright condition without repeated exposure. The frame rate of the videos is about ×0.3 of the actual one (MP4, 380 KB [URL: https://doi.org/10.1117/1.JBO.29.1.016006.s1]).

**Video 2.** Obtained $c_{\mathrm{tHb}}$ and ${\mathrm{StO}}_{2}$ maps video of subject #1’s volar forearm in bright condition with exposures repeated three times. The frame rate of the videos is about ×0.3 of the actual one (MP4, 518 KB [URL: https://doi.org/10.1117/1.JBO.29.1.016006.s2]).

## Data Availability

Data and code developed in this study are available upon reasonable request to the corresponding author.
